# Population survey of the prevalence, impact and care of foot problems in people with rheumatoid arthritis

**DOI:** 10.1186/1757-1146-8-S1-A9

**Published:** 2015-04-20

**Authors:** Oonagh Wilson, Sarah Hewlett, Jon Pollock, Jim Woodburn, Enid Quest, Caroline Swales, John Kirwan

**Affiliations:** 1 Faculty of Health and Applied Sciences, University of the West of England, Bristol, UK; 2 Institute for Applied Health Research, Glasgow Caledonian University, Glasgow UK; 3 Academic Rheumatology Unit, University of Bristol, Bristol, UK

## Aims

Identify the prevalence and impact of foot problems, assess the access to foot care services and describe the care received, in a sample of patients with RA.

## Background

Foot problems in rheumatoid arthritis (RA) derive from a combination of the disease process and altered foot mechanics. Guidelines recommend regular review of patients’ feet but the extent to which the general population of RA patients report foot problems and access foot care has not been established.

## Methods

All RA patients under hospital care in a defined geographical area (Bristol Community Health) were identified. A random sample was sent a postal survey (reminder after 3 weeks) about presence of foot problems, disability (Health Assessment Questionnaire (HAQ)), patient characteristics (age, disease duration, arthritis medication and co-morbidities), and foot care received (if any), including podiatry, orthotics and orthopaedics. Measures of impact (Foot Impact Scale (FIS)) with additional questions (numerical rating scales) related to importance, severity, coping and ability to work derived from a previous study. Socio-economic status was established by IMD scores from postcodes

## Results

Of 739 patients sent the survey, 413 (56%) replied. Responders and non responders were similar for age (63.5 vs.62.6 years), gender (74%F vs. 75%F) and socio-economic status (IMD highest deprivation quintiles 13% vs.15%). Responders’ median (inter-quartile range) disease duration was 10 (5-20) years and HAQ score 1.5 (0.75-2.0). Most responders (394, 95 %) were taking arthritis medication and 273 (66%) reported additional medical conditions (including 28 (7%) with diabetes). Almost all reported current or previous foot problems (n=370, 90%; n=399, 97%, respectively).

Current problems included: articular features 74%; extra articular features 43%; cutaneous lesions 66%; structural deformity 58%; infection 7.5%. Median (IQR) FISIF score 10/21 (6-14); and FISAP score 16/30 (7-23). Median (IQR): importance 6 (3-8); severity 6 (3-8); and coping 5 (3-7). Overall, 38% reported foot problems that affected their ability to work.

Self-care strategies adopted by responders were: aids 188 (46%), cutaneous treatments 268 (65%); CAMs 96 (23%); and devices 275 (67%). A total of 278 (67%) had accessed foot care: podiatry 204 (73%) [Private sector n=149 (54%)]; orthotics 192 (69%); and orthopaedics 92 (32%). Care received included: insoles 190 (66%); prescribed footwear 73 (25%); treatment cutaneous lesions 99 (49%); and foot surgery 72 (35%). Podiatry was the most frequently requested additional service (n=122, 58%)

Relevance/impact: Foot problems are common in patients with RA and impact on many aspects of patients’ lives.

## Outcomes

Unlike previous studies this was representative of all hospital patients with RA and almost all reported foot symptoms. Although FIS scores were slightly lower than in previous studies, substantial impact was reported including affecting ability to work. In spite of this, 30% had never accessed foot care.

## Discussion

Many patients reported current foot problems. Further research is required to compare self-report of foot problems with clinical observations and explore the reasons why patients do and do not access foot care. Also as many patients who had accessed care still reported foot problems, the quality of foot care requires further exploration.

